# Rapidly progressive relapsing polychondritis following surgery for lung adenocarcinoma: a case report

**DOI:** 10.3389/fmed.2025.1629991

**Published:** 2025-10-17

**Authors:** Zhifeng Guo, Linghong Huang, Shaohua Chen, Yunfeng Chen

**Affiliations:** ^1^Department of Respiratory Pulmonary and Critical Care Medicine, The Second Affiliated Hospital of Fujian Medical University, Quanzhou, China; ^2^Department of Endocrinology, The Second Affiliated Hospital of Fujian Medical University, Quanzhou, China; ^3^Department of Pathology, The Second Affiliated Hospital of Fujian Medical University, Quanzhou, China

**Keywords:** relapsing polychondritis, lung adenocarcinoma, postoperative progression, autoimmune disease, case report

## Abstract

**Background:**

Relapsing polychondritis (RP) is a rare autoimmune disease. This paper reports a unique case of RP with rapid progression after surgery for lung adenocarcinoma.

**Case presentation:**

A 75-year-old man presented with a right upper lung mass on physical examination, which was finally diagnosed as invasive adenocarcinoma (stage IB) by pathology, and underwent thoracoscopic lobectomy without further chemotherapy or immunotherapy. Within 90 days after surgery, the patient developed shortness of breath, cough, and redness and swelling of the ear, and he was subsequently diagnosed with RP after pathological examination of the auricular cartilage. The patient experienced temporary improvement with mechanical ventilation, methylprednisolone, and intravenous immunoglobulin therapy, during which time comprehensive rehabilitation was initiated. However, he subsequently developed upper gastrointestinal bleeding, polymicrobial infections, and hemophagocytic syndrome, which proved fatal.

**Conclusion:**

This case highlights the rare temporal correlation of RP as a paraneoplastic syndrome that can occur prior to or concurrently with tumor onset, but it may also arise following tumor resection. Because of the rarity and adverse consequences of RP, clinicians should improve their recognition of the disease to permit early diagnosis and treatment.

## Introduction

Relapsing polychondritis (RP) is a rare autoimmune disease characterized by recurrent inflammation and destruction of cartilaginous tissues, often affecting the ears, nose, and airways (1–4). Its clinical symptoms include red, swollen, painful auricles, as well as difficulty breathing. The etiology of RP is unclear, the existing literature suggests that the pathogenesis of RP might be related to alterations in the tumor microenvironment and immune reconstitution (5).

Lung adenocarcinoma is the most common type of lung cancer, which common treatment methods include surgical resection, chemotherapy, and immunotherapy. In patients with lung adenocarcinoma, postoperative complications such as RP can lead to severe consequences such as airway collapse; therefore, monitoring and follow-up of patients after surgery are crucial (6–8).

RP has complex associations with a variety of malignancies, often appearing as paraneoplastic syndromes that can precede or coincide with tumorigenesis (8–10). To date, few case reports have documented the association between pulmonary malignancies and RP. Yuan et al. and Arakawa et al. both reported case reports of lung cancer complicated by RP (6, 8). The most frequently reported malignancies associated with RP are hematological disorders, particularly myelodysplastic syndromes (MDS). Hebbar et al. documented five cases of concurrent MDS and RP while also reviewing 10 previously reported cases, suggesting a non-coincidental relationship between these conditions. They proposed that immune dysregulation secondary to MDS might play a pivotal role in the pathogenesis of RP (11). Conversely, reports linking RP to solid tumors remain exceedingly rare. For instance, Mase et al. reported a patient who developed RP following esophageal cancer surgery and chemotherapy (9). Ogimoto et al. described a patient who developed RP during nivolumab therapy for mandibular cancer (12). Similarly, Mutoh et al. documented a patient who developed painful bilateral auricular swelling 120 days after pembrolizumab treatment for squamous cell carcinoma of the lower lip (13). Notably, to our knowledge, no cases of RP occurring after surgery for a pulmonary malignancy have been reported.

This case report presents a case of RP that was notable for its rapid progression shortly after tumor resection. This suggests that RP should be considered a potential complication in patients undergoing surgery for lung adenocarcinoma to permit timely intervention and treatment.

## Case presentation

This case report describes a 75-year-old man who presented with a “right upper lung mass” during a routine examination 180 days before the diagnosis of RP. The patient had been previously healthy, with no history of hypertension, diabetes, cardiovascular disease, or chronic pulmonary disorders. There was no personal or family history of autoimmune diseases (including rheumatoid arthritis, systemic lupus erythematosus, vasculitis, or thyroid disorders) and no documented malignancies among first-degree relatives. He had no known drug allergies. He had never smoked, and consumed alcohol only socially. He underwent thoracoscopic right upper lobectomy and mediastinal lymphadenectomy. Histopathology revealed invasive adenocarcinoma (stage IB, pTNM T2aN0M0) with immunoprofile TTF-1(+), Napsin-A(+), CK7(+), CK5/6(−), P40(−), Ki67 10%, Syn(−), CK20(−), consistent with pulmonary origin. PD-L1 immunostaining was not performed. Postoperative assessments were conducted to evaluate inflammatory, tumor, and immunological indices (Table 1). All inflammatory markers, immunological parameters and tumor markers remained within their respective reference ranges, revealing no clinically significant or disease-specific abnormalities. No chemotherapy or immunotherapy was administered after surgery.

**TABLE 1:** Laboratory data.

Imflammatory markers			Immunology		
Characteristics	Value	Reference range	Characteristics	Value	Reference range
WBC (10^∧^9/L)	10.12 ↑	3.50–9.50	LAC-SR	1.11	≤1.2
NE (10^∧^9/L)	6.77	1.80–6.30	LAC-C(s)	35.4	30–38
LY (10^∧^9/L)	2.47	1.10–3.20	LAC-CR	1.07	≤1.2
PCT (ng/mL)	0.11 ↑	0.00–0.05	LAC-R	1.04	≤1.2
CRP (mg/L)	10.08 ↑	0.00–6.00	**Cytokine**		
ESR (mm/H)	20 ↑	≤15	**Characteristics**	**Value**	**Reference range**
**Immunology**			IL-2(pg/mL)	1.22	≤7.5
**Characteristics**	**Value**	**Reference range**	IL-4(pg/mL)	1.6	≤8.56
RF (IU/mL)	9.25	0–14	IL-6(pg/mL)	38.45 ↑	≤5.4
Anti-ccp (U/mL)	0.5	0–5	IL-10(pg/mL)	3.98	≤12.9
MPO-IgG (AU/mL)	2.1	<16	TNF-α(pg/mL)	1.9	≤16.5
PR3-IgG (AU/mL)	2.9	<16	IFN-γ(pg/mL)	2.23	≤23.1
P-ANCA	(−)	(−)	IL-1β(pg/mL)	0.93	≤12.4
C-ANCA	(−)	(−)	IL-5(pg/mL)	0.51	≤3.1
GBM-IgG (AU/mL)	2	<16	IL-8(pg/mL)	21.22 ↑	≤20.6
ANA nuclear dots	(−)	(−)	IL-12p70(pg/mL)	0.21	≤3.4
ANA homogeneous	(−)	(−)	IL-17(pg/mL)	1.21	≤21.4
ANA peripheral	(−)	(−)	IFN-α(pg/mL)	0.58	≤8.5
ANA nucleolar	(−)	(−)	**Tumor markers**		
ANA speckled	(−)	(−)	**Characteristics**	**Value**	**Reference range**
ANA cytoplasmic	(+)1:100	(−)	ProGRP (pg/mL)	66.75	0–68.3
ANA centromere	(−)	(−)	AFP (ng/mL)	2.17	0–7
PM-Scl (AI)	0.05	<1	CEA (ng/mL)	4.67	0–5
PCNA (AI)	0.01	<1	CA19-9 (U/mL)	7.11	0–30
AMA-M2 (AI)	0.11	<1	CA72-4 (U/mL)	1.73	<6.9
U1-snRNP (AI)	0.08	<1	Cyfra21-1 (ng/mL)	3.33 ↑	<3.3
Sm (AI)	0.01	<1	NSE (ng/mL)	13.45	<16.3
SS-A (AI)	0.02	<1	fPSA(ng/mL)	0.435	0–1.5
RO52 (AI)	0.85	<1	tPSA(ng/mL)	3.03	<4
SS-B (AI)	0.09	<1	fPSA/tPSA	0.14 ↓	>0.16
Scl-70 (AI)	0.04	<1	ProGRP(pg/mL)	58.93	0–68.3
Jo (AI)	0.02	<1	**Regulatory T Cells**		
CB (AI)	0.02	<1	**Characteristics**	**Value**	**Reference range**
DNA (IU/mL)	10	<100	CD3+%(%)	57.15	50.62–85.22
NUC (AI)	0.01	<1	CD3+CD4+%(%)	34.74	20.17–58.99
Hi (AI)	0.01	<1	CD3+CD8+%(%)	21.32	8.99–36.72
P0(AI)	0.01	<1	CD4+/CD8+	1.63	1.4–2.0
ACL-A(RU/mL)	1.41	0–20	CD3-CD19+%(%)	11.09	6.3–20.8
ACL-M(RU/mL)	1.08	0–20	CD3-CD56+CD16+%(%)	31.49	3.06–36.21
ACL-G(RU/mL)	7.76	0–20	CD3+(/uL)	1020.22	520–2860
**Immunology**			**Regulatory T Cells**		
**Characteristics**	**Value**	**Reference range**	**Characteristics**	**Value**	**Reference range**
β2-GP1-A(AU/mL)	262.38↑	<16	CD3+CD4+(/uL)	620.07	400–1610
β2-GP1-M(AU/mL)	2.29	<16	CD3+CD8+(/uL)	380.6	150–1250
β2-GP1-G(AU/mL)	13.73	<16	CD3-CD19+(/uL)	197.94	70–620
LACS(s)	36.5	31–44	CD3-CD56+CD16+(/uL)	562.04	80–900
			CD45+Lym#(/uL)	1785.08	1230–3700

WBC, white blood cell; NE, neutrophil; LY, Lymphocyte; PCT, procalcitonin; CRP, C-reactive protein; ESR, erythrocyte sedimentation rate; RF, rheumatoid factor; Anti-CCP, anti-cyclic citrullinated peptide antibody; MPO-IgG, myeloperoxidase IgG antibody; PR3-IgG, proteinase 3 IgG antibody; P-ANCA, perinuclear anti-neutrophil cytoplasmic antibody; C-ANCA, cytoplasmic anti-neutrophil cytoplasmic antibody; GBM-IgG, glomerular basement membrane IgG antibody; ANA, antinuclear antibody; PM-Scl, PM-Scl antibody; PCNA, proliferating cell nuclear antigen antibody; AMA-M2, anti-mitochondrial antibody M2; U1-SnRNP, U1 small nuclear ribonucleoprotein antibody; Sm, smith antibody; SS-A, Sjögren’s syndrome; Ro52, Ro52 antibody; Scl-70, anti-topoisomerase I antibody; Jo-1, anti-Jo-1 antibody; CB, anti-centromere B antibody; DNA, anti-dsDNA, NUC, anti-nucleosome; Hi, anti-histone antibody; ACL, anti-cardiolipin antibody; β2-GP1, anti-β2 glycoprotein 1; LACS/SR/C/CR/R, lupus anticoagulant screen/confirm/ratio; IL, interleukin; TNF, tumor necrosis factor; IFN, interferon; ProGRP, pro-gastrin-releasing peptide; AFP, alpha-fetoprotein; CEA, carcinoembryonic antigen; CA, carbohydrate antigen; Cyfra21-1, cytokeratin 19 fragment 21-1; NSE, neuron-specific enolase; fPSA, free prostate-specific antigen; tPSA, total prostate-specific antigen; CD, cluster of differentiation; Treg, regulatory T cells.

**FIGURE 1:**
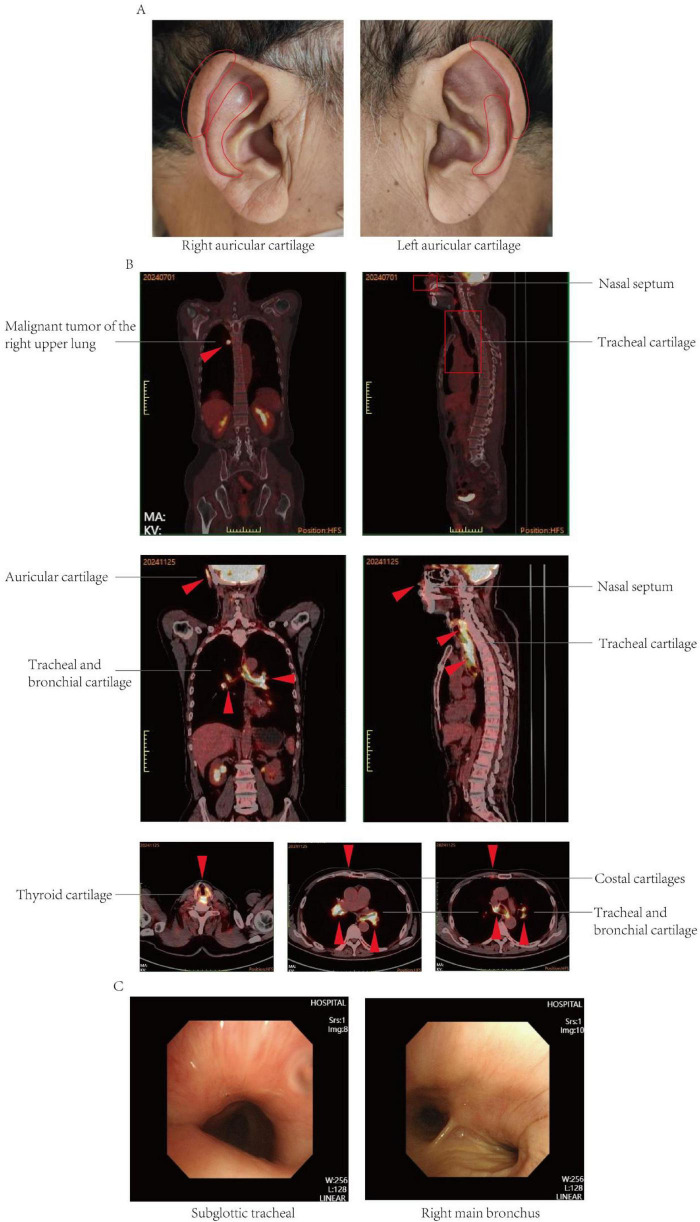
**(A)** Clinical pictures at presentation. Note the distinctive swelling and redness of the right auricular cartilage and left auricular cartilage. **(B)** 18F-FDG PETCT imaging. Preoperative 18F-FDG PET/CT imaging revealed a nodular lesion in the right upper lobe of the lung exhibiting increased metabolic activity, suggestive of a malignant tumor. The preoperative 18F-FDG PET/CT imaging did not demonstrate increased metabolic activity in the nasal septal and tracheal cartilage. Postoperative 18F-FDG PETCT showed that the density of cricoid cartilage was higher than before, and the walls of trachea and bronchus were abnormally thick and dense, and the nasal septum, bilateral auricular cartilage, bilateral thyroid cartilage, and bilateral costal cartilage were with diffuse abnormal FDG metabolism, which was considered relapsing polychondritis. **(C)** Bronchoscopy of tracheal stenosis in present case report. The procedure revealed a subglottic tracheal collapse accompanied by mucosal hyperplasia; the right main bronchus exhibited signs of softening and collapse.

A duration of 90 days postoperatively, the patient developed redness, swelling, and pain in the auricle, along with fever (37.8 °C) (Figure 1A). Anti-infective and anti-inflammatory therapy did not improve the patient’s condition. A duration of 210 days after surgery, the patient experienced progressively worsening dyspnea, accompanied by cough and sputum production, with the sputum being scant and white, but no findings of chest pain, hemoptysis, abdominal distension, abdominal pain, acid reflux, or heartburn. His dyspnea progressively worsened.

Follow-up PET-CT revealed increased density of the cartilaginous structures; abnormal thickening and increased density of the tracheal and bronchial walls; and diffuse metabolic abnormalities in the nasal septum, bilateral auricular cartilage, bilateral thyroid cartilage, and multiple costal cartilages, which were considered benign and possibly indicative of RP. A review of the preoperative PET-CT findings did not reveal any corresponding areas of hypermetabolism, and the intraoperative pathology did not uncover signs of polychondritis (Figure 1B).

A clinical diagnosis of RP is typically based on the criteria initially proposed by McAdam et al. (14) and later modified by Damiani and Levine (15), with alternative criteria established by Michet et al. (16) (Supplementary Table 1). In the present case, PET-CT demonstrated involvement of the auricular, nasal, and tracheal cartilage, and auricular biopsy confirmed relapsing polychondritis; thus, the McAdam, Damiani-Levine, and Michet diagnostic criteria were satisfied.

**FIGURE 2:**
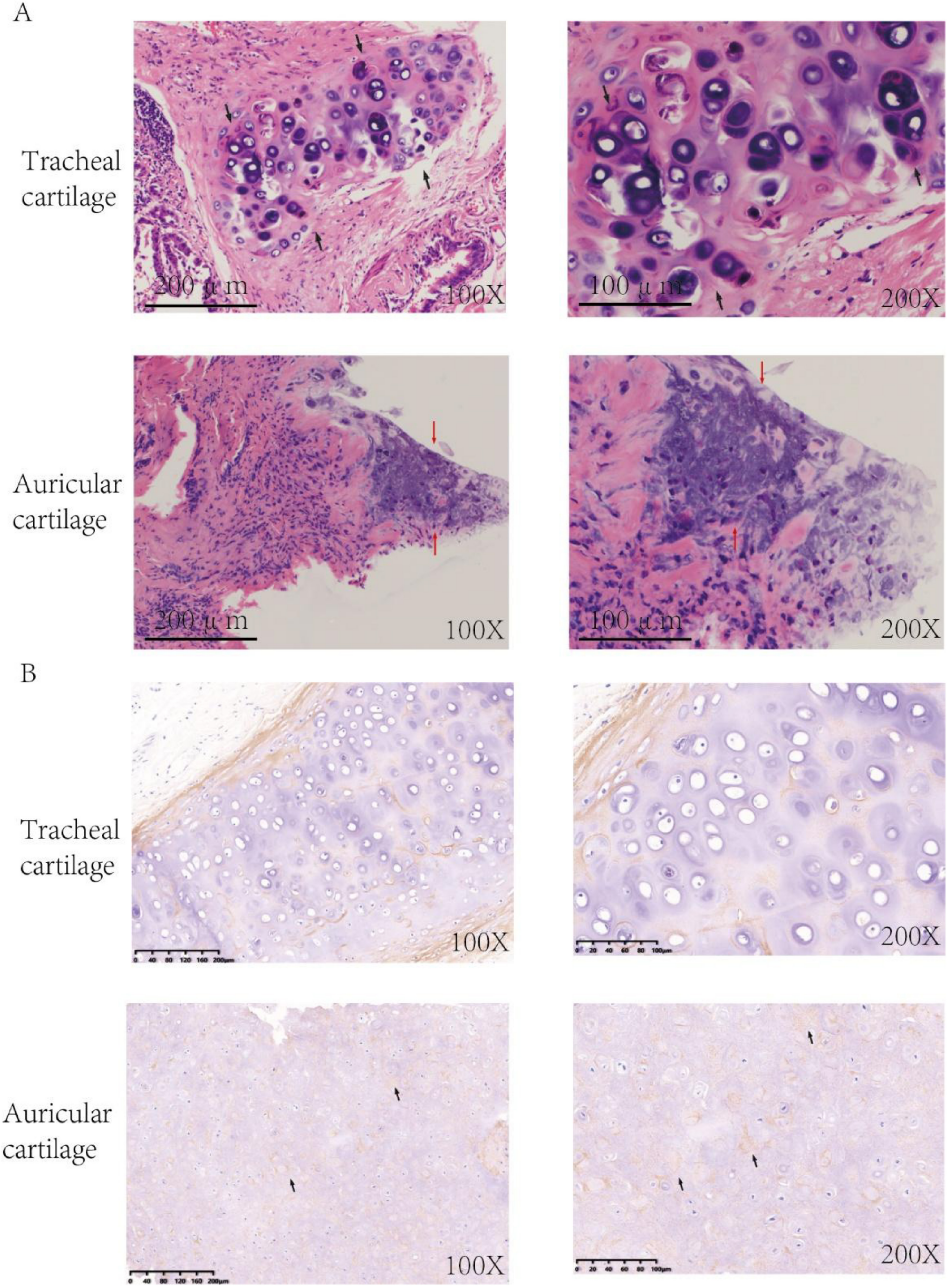
**(A)** HE-stained sections of cartilage tissue. HE staining of tracheal cartilage prior to surgery is depicted in panels, and HE staining of auricular cartilage tissue post-surgery is depicted (Black arrows: chondrocytes, Red arrows: lymphocyte infiltration). **(B)** Immunohistochemistry of cartilage tissue. Immunohistochemical analysis using Anti-Col II antibody demonstrated significantly elevated expression of the Anti-Col II (black arrows) in postoperative auricular cartilage in comparison to preoperative tracheal cartilage.

A duration of 210 days postoperatively, because of the progression of dyspnea and respiratory failure, the patient underwent endotracheal intubation and mechanical ventilation. Comprehensive laboratory evaluation revealed unremarkable findings, with all measured immunological markers (including rheumatoid factor, antinuclear antibodies, and immunoglobulin profiles) falling within normal reference ranges. Bronchoscopy revealed significant collapse and narrowing of the trachea and left and right main bronchi (Figure 1C). Biopsy of the right ear was performed, and the pathological results revealed the presence of a small amount of cartilage tissue with partial degeneration and necrosis, nuclear pyknosis, reduced basophilia of the cartilage matrix, and fibrous tissue proliferation together with a low level of lymphocytic infiltration, consistent with RP (Figure 2A). Comparative immunohistochemical staining for Anti-Col II was conducted on both the preoperative tracheal cartilage biopsy and postoperative auricular cartilage specimens. Semi-quantitative visual comparison revealed a marked increase in Anti-Col II expression in the auricular cartilage (postoperative sample) relative to that in the tracheal cartilage (preoperative sample) (Figure 2B).

To definitively exclude VEXAS syndrome we systematically compared our patient with the established criteria: (1) demographics–VEXAS occurs almost exclusively in males ≥50 years and our patient was 75 years old; (2) constitutional features–VEXAS patients usually present with recurrent fever and skin involvement, whereas ours had only a single transient low-grade temperature spike (37.8 °C) without sustained fever ≥38.5 °C, rash, or other systemic findings required for diagnosis; (3) hematological abnormalities–macrocytic anemia and/or thrombocytopenia are hallmarks, yet all of our patient’s blood counts remained normal; (4) bone-marrow morphology–cytoplasmic vacuoles in myeloid/erythroid precursors are required, but our biopsy showed only hemophagocytosis without vacuolated precursors; and (5) associated myelodysplasia–∼75% of VEXAS-RP cases have underlying MDS, whereas our patient lacked dysplastic changes. Taken together, the absence of sustained fever≥38.5 °C, cytopenias, vacuolated precursors, and MDS rendered VEXAS syndrome highly improbable; accordingly, since current expert recommendations advise UBA1 sequencing only when ≥2 major VEXAS features are present and our patient fulfilled none, targeted genetic testing was not pursued.

**FIGURE 3:**
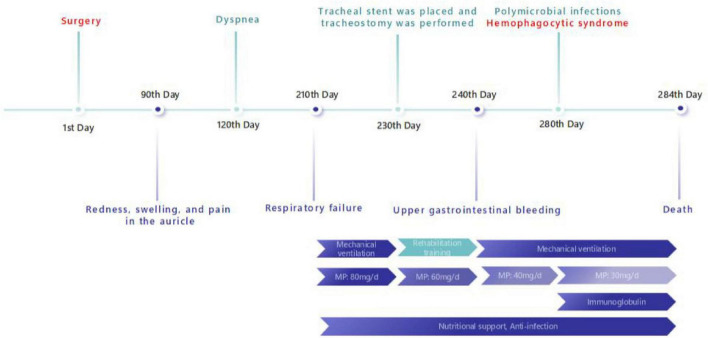
Timeline. A timeline of key points in the patient’ s symptom, diagnosis and treatment in present case report (MP, methylprednisolone).

After being diagnosed with RP, the patient was treated with methylprednisolone 80 mg/day for 20 days. Following treatment, the ventilator support parameters decreased to a level suitable for weaning. However, because of extensive stenosis of the trachea and bilateral main bronchi, the patient had a high risk of recurrent dyspnea upon ventilator withdrawal. Therefore, tracheostomy was performed, and a tracheal stent was placed to provide structural support. Subsequently, the patient was successfully weaned from mechanical ventilation, meanwhile, the methylprednisolone dosage was adjusted to 60 mg/day, and he began rehabilitation training, including whole-body muscle strength training, massage therapy, transfer movement training, and stationary bicycle rehabilitation. Unfortunately, 10 days later, he patient subsequently developed upper gastrointestinal bleeding, upon which we reduced the methylprednisolone dosage to 40 mg/day and managed the bleeding actively. Over the next 40 days, the patient developed multiple infections (bacterial, viral, and fungal), leading us to further taper the methylprednisolone dosage to 30 mg/day; nevertheless, the patient ultimately progressed to hemophagocytic syndrome. Despite aggressive interventions, including ventilator support, broad-spectrum antimicrobial therapy, and intravenous immunoglobulin, the patient’s condition deteriorated, leading to a fatal outcome (Figure 3).

## Discussion

This case describes a 75-year-old man who developed rapidly progressive RP within 90 days following successful resection of right upper lobe invasive adenocarcinoma. The clinical presentation, temporal association, and potential immunological mechanisms included several unique and instructive features. First, although the existing literature describes RP as a paraneoplastic syndrome typically manifesting either before cancer treatment or showing symptom resolution following tumor resection/chemotherapy/immunotherapy, our patient exhibited an opposite clinical course. The rapid onset and progression of RP following curative lung adenocarcinoma surgery with a reduced tumor burden markedly contradicts most reported cases, in which RP improved after cancer treatment. This observation suggests that the tumor exerted an inhibitory effect on RP manifestations, possibly through tumor-associated immunosuppression that masked autoimmune disease activity. Surgical tumor removal might have disrupted this immunosuppressive state, triggering immune system reconstitution or dysregulation that accelerated autoimmune responses, leading to rapid RP progression. This immune reconstitution–autoimmune imbalance hypothesis warrants further investigation. Second, emerging evidence has described posttreatment autoimmune phenomena, including RP and other paraneoplastic autoimmune disorders, following tumor resection, chemotherapy, or immunotherapy. Some researchers term this the immune reconstitution autoimmune response, in which relief from tumor-mediated immunosuppression enhances abnormal self-antigen recognition (14). Our case supports this hypothesis, demonstrating that RP development might be closely correlated with dramatic immune microenvironment changes after treatment, particularly during rapid tumor burden reduction that disrupts immune tolerance. Finally, this case has two main clinical implications; specifically, it alerts clinicians to remain vigilant for rare autoimmune complications such as RP after cancer surgery and provides novel insights into tumor–autoimmune disease interplay. In the era of widespread immunotherapy in particular, treatment-induced immune alterations and their autoimmune consequences merit deeper exploration. In summary, this case’s value lies in its unique temporal pattern and immunological implications, suggesting that the pathogenesis of RP, in addition to the presence of the tumor, might also involve dynamic posttreatment immune changes. Although RP as a paraneoplastic syndrome is documented, our case offers novel clinical and mechanistic perspectives worthy of attention in both research and practice.

Our comprehensive literature search revealed only one reported case of immune-related complications following lung cancer surgery. Ito et al. described an 80-year-old man who developed fever, anorexia, and painful left axillary swelling 60 days after pulmonary adenocarcinoma resection, which was ultimately diagnosed as IgG4-related disease with elevated serum IgG4 levels (15). No other immune complications after lung cancer surgery were identified in the literature. Regarding RP development after malignancies, we identified only one relevant report by Mentzel et al. documenting a 79-year-old man who presented with painful auricular erythema, swelling, conjunctivitis, and mild nasal deformity 3 weeks after squamous cell carcinoma resection, and his condition was later confirmed as RP (16). The literature does feature reports of other autoimmune manifestations following various cancer surgeries. Cases of rheumatoid arthritis, systemic lupus erythematosus, and autoimmune hepatitis onset or exacerbation have been documented after gastric, breast, and colorectal cancer resection (17). These reported cases share remarkable consistency with our present observations, suggesting that dramatic immune system reorganization following cancer treatment can activate previously quiescent autoimmune responses. We therefore propose that RP represents another distinct clinical entity within the spectrum of postoperative immune reconstitution-mediated autoimmune disorders. Compared with Ito et al.’s IgG4-related disease (onset 8 weeks post-lobectomy, serum IgG4 > 1.35 g L^–1^, axillary-node swelling, prompt steroid response) and Mentzel et al.’s RP (auricular pain at 3 weeks, no airway involvement, complete remission on 20 mg prednisolone), our patient developed RP at 12 weeks, exhibited severe tracheobronchial cartilage collapse requiring stenting, and ultimately succumbed to secondary hemophagocytic syndrome despite high-dose immunosuppression. These divergent timelines, organ patterns, and outcomes underscore that RP is a distinct, potentially fulminant member of the postoperative immune-reconstitution spectrum.

The pathogenesis of immune reconstitution inflammatory syndrome (IRIS) is influenced by a multitude of factors, including CD4 + T cells, proinflammatory cytokines, macrophages, and other innate immune cells. Recent research indicates an increase in antigen-specific CD4 + and CD8 + T cells in IRIS patients, alongside a heightened frequency of CD4 + cells expressing programmed cell death protein-1 (PD-1), Ki67, elevated levels of human leukocyte antigen-D related (HLA-DR), and effector memory group markers, which may contribute to the pathogenesis of IRIS (18, 19). Correspondingly, serum levels of interleukin (IL)-6, CXCL-8, tumor necrosis factor (TNF)-α, and interferon (IFN)-γ are significantly elevated in IRIS patients (20, 21). Additionally, innate immune components such as macrophages, monocytes, γδ T cells, natural killer (NK) cells, and the complement system are crucial contributors. Despite numerous reports on IRIS biomarkers, the variability in clinical presentation and the complexity of the disease process continue to constrain the comprehensive understanding of its immune pathogenesis (21–24)

RP is typically associated with malignant tumors, or it might precede the onset of tumor development (6, 8, 25, 26). Prior studies indicated that the occurrence of lung cancer in patients with RP might be related to their autoimmune responses. Research has detected various autoantibodies in patients with RP, and these antibodies might be associated with the development of lung cancer. For instance, elevated levels of Anti-Col II have been correlated with the occurrence of lung cancer (6). Anti-Col II are frequently found in the sera of patients with RP, and the presence of these antibodies is associated with disease activity and severity (6). Furthermore, the inflammatory response in RP might lead to an imbalance in lung tissue damage and repair, thereby creating a favorable microenvironment for tumor development (7). This postoperative delayed onset time pattern suggests that changes in the tumor microenvironment or postoperative immune reconstitution triggered the pathological activity of RP. In patients with RP, the immune system produces an abnormal immune response to self-cartilage components (such as Anti-Col II), leading to the infiltration of inflammatory cells and the release of cytokines, which in turn causes cartilage destruction (7). Additionally, RP might be associated with certain malignancies (such as lung cancer), suggesting that tumors can promote the occurrence of RP by inducing abnormal immune responses (8, 27). Relevant studies illustrated that the immunosuppressive state of tumors might delay the manifestation of RP in the early stages, whereas immune system activation after surgery might accelerate its progression (12, 13).

In present case, the auricular cartilage demonstrated significantly elevated Anti-Col II expression compared with preoperative tracheal cartilage, providing compelling immunohistological evidence supporting the diagnosis of RP. Therefore, it is reasonable to speculate that the temporal association between tumor resection and RP onset suggests a plausible immunopathogenic mechanism. Preoperatively, the tumor microenvironment likely maintained systemic and local immunosuppression through multiple pathways, potentially suppressing autoimmune activity. Surgical tumor removal might have disrupted this immunosuppressive state, leading to the restoration of immune surveillance, altered cytokine profiles, and the redistribution of immune cell populations. These changes could facilitate recognition of previously tolerated self-antigens, particularly cartilage components. This phenomenon aligns with the concept of immune reconstitution, typically observed following the withdrawal of intensive immunosuppression. Emerging evidence suggests that rapid immune system recovery can sometimes precipitate autoimmune disorders through imperfect rebalancing of immune homeostasis (28–30). In our case, the abrupt RP progression following surgery, without intervening chemotherapy or immunotherapy, strongly implicates tumor resection itself as the triggering event for immune microenvironment alterations. The pathophysiology might involve dual mechanisms: unleashing of preexisting but suppressed autoreactive T-cells and antibodies against cartilage antigens and neo-sensitization to self-antigens during immune reconstitution. This is particularly relevant given RP’s established pathogenesis involving both humoral and cell-mediated immunity against connective tissues. Specifically, this case underscores several critical clinical implications: the necessity for vigilant monitoring of autoimmune manifestations post-surgery, the detection of autoimmune complications, and the identification of potential biomarkers in patients exhibiting elevated levels. For patients undergoing curative-intent lung cancer resection who are at potential risk of RP, we propose the following pragmatic monitoring protocol: (1) Symptom screen every 2 weeks for the first 90 days: use a simple 5-item checklist (auricular pain/swelling, nasal chondritis, ocular inflammation, arthralgia, dyspnea). (2) Laboratory panel at 2, 6 and 12 weeks: CBC with differential, CRP, ESR and baseline ANA; any new cytopenia or rising CRP should trigger rheumatology referral. (3) Imaging: low-dose chest CT at 4 weeks post-op to establish an airway baseline; if new onset dyspnea or cough appears, add high-resolution CT or PET-CT to detect subglottic or bronchial wall thickening. (4) Early specialist involvement: otolaryngology or rheumatology consultation within 72 h if any two items from the symptom checklist are positive. (5) Biomarker consideration: although not yet validated for routine use, serum Anti-Col II antibodies can be measured if available; a ≥2-fold increase above baseline warrants further evaluation. This concise, step-wise approach allows prompt recognition and initiation of immunomodulatory therapy before airway compromise occurs. It is essential to recognize that tumor resection may act as a catalyst for immune dysregulation. Emphasizing early diagnosis and intervention in analogous cases is crucial to prevent the recurrence of similar complications. Furthermore, this case provides valuable insights into cancer immunology research and enhances our understanding of the bidirectional relationship between malignant tumors and autoimmunity. Our findings contribute to growing evidence of autoimmune syndromes after tumor resection and emphasize the delicate balance between tumor-induced immunosuppression and postoperative immune reconstitution. Further research should explore predictive biomarkers and preventive strategies for high-risk patients undergoing cancer surgery.

The dynamic changes monitored by PET-CT are of significant importance in assessing the progression of RP (7, 31). Previous case reports documented instances in which RP was misdiagnosed as lung cancer (32). In this case, preoperative PET-CT only revealed tumor lesions without any inflammatory manifestations of RP, which might be related to the subclinical state of early RP or tumor-associated immunosuppression. Postoperative PET-CT demonstrated widespread chondritis, suggesting that immune system activation following tumor resection might have accelerated the progression of RP. Prior research indicated that patients with lung cancer who underwent treatment with PD-1 blockade experienced respiratory distress, with subsequent PET-CT examinations revealing thickening and abnormal FDG uptake in the tracheobronchial and nasal septum cartilage, ultimately leading to a diagnosis of RP (12). This dynamic imaging evolution provides important insights into the pathological mechanisms of RP and underscores the potential value of imaging surveillance in the early detection of complications (5).

The patient presented without typical signs of respiratory infection (e.g., purulent sputum) and exhibited negative results in rheumatologic screening tests. Follow-up CT revealed no new infiltrates, cavitary lesions, or evidence of tumor recurrence/metastasis. Importantly, the patient had not received postoperative radiotherapy, chemotherapy, or immune checkpoint inhibitors, nor did he have a history of corticosteroid or immunomodulatory drug use before or after surgery. These findings collectively eliminated infectious processes, disease recurrence, drug-induced reactions, and other rheumatologic conditions as potential causes of the patient’s respiratory symptoms and chondritis. The diagnosis of RP was confirmed by characteristic clinical features including auricular erythema/swelling, airway involvement with bilateral symmetrical lesions, nasal cartilage involvement, and histopathological evidence of lymphocytic infiltration and cartilage matrix destruction on ear cartilage biopsy. The subsequent marked clinical improvement following methylprednisolone therapy further supported the definitive diagnosis of RP.

The rapid progression of RP can lead to severe complications (5). However, reports of such rapid deterioration in the literature are relatively rare, suggesting that clinicians should remain vigilant for the explosive course of RP in patients following tumor surgery and emphasize the importance of early intervention. This characteristic both affects patient prognosis and provides a new warning signal for clinicians. RP is associated with a high mortality rate, particularly in cases involving airway involvement (4). After the diagnosis was established, the patient promptly received high-dose methylprednisolone, which initially improved his airway inflammation. Subsequently, however, glucocorticoid-related complications–upper-gastrointestinal bleeding followed by polymicrobial (bacterial, viral and fungal) infections–developed. These overwhelming infections triggered hemophagocytic syndrome, which became the immediate and direct cause of death. The existing literature suggests that chemotherapy or immunotherapy can improve paraneoplastic RP (2, 8). However, in this case, the deterioration of RP occurred even after tumor resection, indicating the necessity of postoperative immunomodulatory strategies, such as corticosteroids or biological agents. This contradictory phenomenon warrants further investigation to provide insights for future treatment protocols. The patient’s eventual death, despite an initial positive response to steroid treatment, highlights the complexity of managing RP. The challenges are magnified when the condition is complicated by iatrogenic immunosuppression and secondary hemophagocytic syndrome.

In addition, PET-CT revealed the involvement of multiple cartilaginous structures, including the nasal septum. Such an abrupt development of autoimmune disease is clinically uncommon. The existing literature suggests that cancer treatment, particularly surgical resection, can profoundly disrupt immune homeostasis, potentially triggering previously suppressed autoimmune responses, a phenomenon termed immune reconstitution autoimmune disease (33). Our case might represent an illustrative example of this entity. Although the existence of subclinical RP prior to surgery cannot be entirely excluded, the absence of prodromal symptoms (e.g., otolaryngologic complaints, arthralgia, ocular involvement, systemic inflammation) and unremarkable preoperative imaging/laboratory findings make preexisting occult RP unlikely. Moreover, the patient’s initial postoperative recovery was uneventful, with no signs of acute immune activation until the abrupt emergence of RP symptoms after 90 days, supporting the hypothesis of *de novo* postoperative onset or rapid progression. Multiple perioperative factors, including anesthetic agents, surgical trauma, inflammatory stress, and the release of autoantigens from damaged tissues, could contribute to immune dysregulation. Notably, the systemic inflammatory response and subsequent immunomodulatory shifts following surgery can disrupt preexisting immune tolerance, thereby precipitating autoimmune disease. Although no single mechanism has been definitively established, these insights offer potential research directions for understanding postoperative RP. Although a causal relationship between lung adenocarcinoma resection and RP cannot be confirmed in this isolated case, the striking temporal association and postoperative immunological alterations suggest a plausible pathophysiological link worthy of further investigation.

This study presents several noteworthy limitations that should be considered when interpreting the findings. Primarily, as a single-case report, the observations are inherently influenced by individual biological variability and lack a control cohort, thereby limiting the generalizability of the conclusions. From a methodological perspective, three specific constraints merit emphasis: first, the absence of UBA1 genetic sequencing prevents molecular confirmation of the suspected VEXAS syndrome pathogenesis; second, the lack of comprehensive dynamic immune monitoring throughout the clinical course restricts our understanding of the temporal relationship between immune reconstitution and disease manifestations; third, the inherent heterogeneity of obtained cartilage tissue samples introduces potential interpretation biases in histological assessments.

Building upon these findings, we propose a structured research agenda to address the identified gaps. Future investigations should prioritize: first, implementing prospective studies incorporating serial immune monitoring in the peri-operative period to delineate the dynamics of immune reconstitution following major surgery; second, establishing systematic UBA1 mutation screening protocols for patients presenting with overlapping rheumatologic-hematologic syndromes, which would enhance early diagnosis of VEXAS spectrum disorders; third, developing standardized immunohistochemical evaluation frameworks for cartilage tissue analysis, incorporating appropriate control samples to improve reproducibility and comparative analysis across studies. These directed approaches would substantially advance our understanding of immune dysregulation in similar clinical contexts and facilitate the development of targeted diagnostic and therapeutic strategies.

## Conclusion

This case provides new insights into the complex relationship between RP and malignant tumors and highlights the impact of the postoperative immune status on the rapid progression of RP. Future research should further explore the pathogenesis of RP and investigate effective monitoring and intervention strategies in patients with cancer.

## Data Availability

The original contributions presented in this study are included in this article/Supplementary material, further inquiries can be directed to the corresponding author.
